# Resveratrol alleviates testicular toxicity induced by anti-PD-1 through regulating the NRF2-SLC7A11-GPX4 pathway

**DOI:** 10.3389/fimmu.2025.1529991

**Published:** 2025-03-12

**Authors:** Halahati· Tuerxun, Yixin Zhao, Yawen Li, Xingyu Liu, Shuhui Wen, Yuguang Zhao

**Affiliations:** Cancer Center, The First Hospital of Jilin University, Changchun, Jilin, China

**Keywords:** immune checkpoint inhibitors, anti-programmed cell death protein 1, reproductive toxicity, oxidative stress, ferroptosis, resveratrol

## Abstract

**Background:**

Fertility preservation is a critical concern for reproductive-age cancer survivors, as conventional cytotoxic therapies can cause irreversible damage to the reproductive system, potentially depriving them of the ability to have children in the future. Immune checkpoint inhibitors (ICIs), including anti-programmed cell death protein 1 (anti-PD-1), have become a standard therapeutic approach for various malignancies. However, the impact of ICIs on reproductive function and fertility is not well understood and remains a largely unexplored domain. Resveratrol (RSV), a plant-derived compound, has shown potential as an nuclear factor erythroid 2-related factor 2 (NRF2) agonist to counteract reproductive toxicity induced by various diseases, drugs, and environmental toxins.

**Methods:**

Male C57BL6/J mice with B16 melanoma were assigned into four groups. RSV and ICI/RSV groups received RSV (40 mg/kg) orally every other day for one month, while controls received the vehicle. ICI and ICI/RSV groups were injected with anti-PD-1 antibody (10 mg/kg) weekly, and controls received IgG2b kappa antibody. Parameters like body and testicular weight, sperm concentration, and western blot for ferroptosis markers were measured. Furthermore, oxidative stress biomarkers, lipid oxidation factors, and gonadal hormone levels were quantified using commercial kits.

**Results:**

Anti-PD-1 therapy caused male reproductive dysfunction, as evidenced by reduced sperm concentration, altered gonadal hormone levels, and disruption of blood-testis barrier (BTB) integrity. Furthermore, ferroptosis was a key mechanism in anti-PD-1-induced testicular dysfunction, characterized by disrupted iron homeostasis, elevated lipid peroxidation, and suppression of the system Xc−/glutathione peroxidase 4 (GPX4) axis. Additionally, anti-PD-1 therapy diminished antioxidant defenses by inhibiting the NRF2 pathway, thereby increasing the susceptibility to ferroptosis. Crucially, RSV treatment ameliorated anti-PD-1-induced reproductive dysfunction. This was achieved by reducing T cell infiltration, lowering interferon-gamma levels, activating the NRF2 pathway, and maintaining iron and lipid homeostasis.

**Conclusions:**

Our study demonstrates that anti-PD-1 triggers oxidative stress and ferroptosis in the testis, causing male reproductive dysfunction. RSV may offer protection against testicular toxicity associated with anti-PD-1, particularly through its antioxidant and anti-ferroptosis properties.

## 1 Introduction

With the continuous refinement of cancer therapies, survival rates have notably improved, particularly among individuals of reproductive age (1). While cytotoxic radiotherapy and chemotherapy remain the mainstay of cancer treatments, their systemic side effects cannot be overlooked (2). In light of their limitations, there is a growing search for alternative treatments that may offer similar therapeutic benefits with fewer detrimental side effects. Immune checkpoint inhibitors (ICIs), which target immune checkpoint regulators such as programmed cell death protein 1 (PD-1), its ligand PD-L1, or cytotoxic T lymphocyte-associated antigen 4 (CTLA-4), have emerged as a promising alternative by enhancing the activation and proliferation of tumor-specific T cells.

However, the use of ICIs has also been associated with a range of immune-related adverse events (irAEs), including well-documented toxicities such as gastrointestinal, cardiac, hepatic, and dermal effects (3–6), as well as potential impacts on reproductive function (7, 8). This latter aspect, which is less studied but crucial for patients of reproductive age, forms a significant focus of our investigation. Given the widespread use of ICIs among cancer patients of reproductive age (9, 10), the potential impact on reproductive health is a pressing concern that has received limited attention. While recent studies have demonstrated that ICIs can impair ovarian function in female mice (11, 12), research on the mechanisms by which ICIs affect the male reproductive system remains scarce. Therefore, understanding the toxic effects of ICIs on male reproduction is both critical and timely, highlighting the need for further investigation in this area.

The homeostatic regulation of iron is crucial for testicular function, playing a significant role in the biosynthesis of testosterone and the intricate mechanisms of spermatogenesis (13, 14). However, excessive iron can induce oxidative stress and lipid peroxidation, potentially leading to ferroptosis—an iron-dependent form of regulated cell death characterized by the accumulation of lipid peroxides and the depletion of glutathione peroxidase 4 (GPX4) (15–17). The solute carrier family 7 member 11 (SLC7A11)-glutathione (GSH)-GPX4 axis is believed to constitute the major system countering ferroptosis (18–21). Notably, the redox imbalance, resulting from an overproduction of oxidants or a deficiency of antioxidants, is a key driver of ferroptosis (22), which is increasingly recognized as a cause of reproductive system impairment. GPX4 is particularly important for germ cell health, with lower levels associated with oligospermia-related infertility (23–25). In murine models, GPX4 gene knockout has resulted in reduced sperm concentration and male infertility (26). Moreover, reduced expression of GPX4 and SLC7A11 in individuals with asthenospermia correlates with increased ferroptosis and impaired sperm function (24). Given that testicular exposure to certain toxins and drugs can trigger ferroptosis (14, 27–29), targeted suppression of this process may be a viable strategy for protecting testicular health. However, it is currently unclear whether ICIs contribute to male reproductive impairment by inducing ferroptosis in the testis.

Nuclear factor erythroid 2-related factor 2 (NRF2) is an antioxidant transcription factor crucial for maintaining redox balance (30–33). Notably, downstream target genes of NRF2, such as heme oxygenase-1 (HO-1) and ferritin, are closely related to iron homeostasis (34). Furthermore, the transferrin receptor (TFR) and ferroportin (FPN), key components of iron metabolism on the cell membrane are both regulated by NRF2 (35, 36). In addition, NRF2 influences the synthesis and metabolism of the GSH and related enzymes (33, 37), as well as the cystine/glutamate antiporter (system Xc-), which is upstream of the GSH-GPX4 axis (33, 38, 39). Therefore, given its regulatory role in iron homeostasis and the inhibition of lipid peroxidation, NRF2 is acknowledged as a pivotal mechanism for countering ferroptosis (33–39). Moreover, clinical studies have consistently demonstrated a significant reduction in NRF2 expression level within the sperm of oligospermic individuals, which correlates with oxidative stress and subsequent disruption of spermatogenesis (40–43).

Resveratrol (RSV), a polyphenolic compound predominantly found in grapes and wine, exhibits a diverse array of pharmacological attributes (44). Its spectrum of activities includes potent antioxidant, anti-inflammatory, anti-aging, and anti-neoplastic effects, complemented by anti-apoptosis and immunomodulatory capabilities, as well as its potential in modulating addictive behaviors (44–46). These multifaceted therapeutic potentials highlight RSV as a promising candidate for various medical applications. Of particular interest is the emerging role of RSV in attenuating organ toxicity, which is believed to be mediated through its anti-ferroptosis properties. For example, RSV has been demonstrated to protect BEAS-2B cells from Erastin-induced ferroptosis by modulating the NRF2-Kelch-like ECH-associated protein 1 (KEAP1) pathway (47). Additionally, it has mitigated cardiotoxicity associated with 5-FU by inhibiting ferroptosis in a GPX4-dependent manner (48), and has been implicated in neuroprotection through the activation of the NRF2-GPX4 axis (49). The influence of RSV on the expression of key ferroptosis-related genes, such as NRF2, SLC7A11, and GPX4, emphasizes its potential regulatory role in the ferroptosis process (50, 51). More importantly, our previous research, along with extensive studies, has demonstrated RSV’s positive effects as an NRF2 agonist in countering reproductive toxicity induced by various diseases, drugs, and environmental toxins (32, 52–55). However, the potential of RSV to protect against ICIs-induced male reproductive toxicity requires further investigation.

The objective of the current study is to investigate the effects of anti-PD-1 on male reproductive system, with a specific focus on oxidative stress and ferroptosis. Furthermore, we aim to explore whether RSV supplementation is effective in mitigating the detrimental effects caused by anti-PD-1 on male fertility, providing insights into protective strategies against ICIs-induced reproductive toxicity.

## 2 Materials and methods

### 2.1 Animals and treatment

In compliance with the National Institutes of Health Guidelines for the Care and Use of Laboratory Animals, all animal experimental procedures were reviewed and approved by the Animal Ethics Committee of Jilin University. The study utilized eight-week-old male C57BL6/J mice (Weitonglihua, Beijing, China), which were allowed to acclimate under standardized conditions in a temperature-controlled environment set at 22°C with a 12:12-hour light-dark cycle. The animals had ad libitum access to standard rodent chow and tap water.

Mouse B16 melanoma cells were obtained from the China Center for Type Culture Collection (CCTCC, China) and cultured in RPMI 1640 medium (Catalog No. 10491, Solarbio, Beijing, China), 10% fetal bovine serum (FBS) (Catalog No. ASFBS-U, Assay Matrix), and penicillin/streptomycin. Cells were resuspended in phosphate-buffered saline (PBS) at a concentration of 2×10^5^ cells per ml and then subcutaneously injected in a final volume of 50μl into each mouse. Once palpable, tumors were measured with digital calipers, and tumor volume was calculated using the formula: (length×width^2^)/2 = volume (mm³). The maximum tumor volume allowed by the ethics committee was 1,500 mm³, and tumors never exceeded this limit. Once tumors reached approximately ~30 mm³, mice were randomized into four distinct groups, each comprising seven individuals: control group, anti-PD-1 (ICI) treatment group, RSV treatment group, anti-PD-1 with RSV (ICI/RSV) treatment group. Carboxy methylcellulose (CMC) (C8621, Beijing, China) at a concentration of 0.01% was employed as the vehicle for RSV. Mice in the RSV and ICI/RSV groups were administered RSV (Sigma Aldrich, St. Louis, MO, USA) via gavage at a dosage of 40 mg/kg on alternate days over a one-month period (32). The control and ICI groups received an equivalent volume of 0.01% CMC. The ICI and ICI/RSV groups were injected intraperitoneally with anti-mouse PD-1 (Selleck, China) at a dosage of 10 mg/kg once weekly for a duration of one month. Concurrently, the control and RSV groups received an equivalent volume of antibody IgG2b kappa (BioXcell).

At the conclusion of the experimental phase, the mice were humanely euthanized under deep anesthesia. Blood, along with tissues from the bilateral testes, epididymides, and spleens, were meticulously collected for subsequent scientific analyses.

### 2.2 Assessment of sperm parameter

Sperm samples were extracted from the cauda epididymides and immediately immersed in isotonic saline maintained at a temperature of 37°C. The concentration of spermatozoa within the epididymal fluid was quantified using a hemocytometer, a standard method for cell counting (56). The procedure involved a meticulous dissection of the epididymis and cauda epididymis using fine scissors, followed by their transfer into a petri dish containing 1 ml of pre-warmed isotonic saline. The cauda epididymis was then carefully incised 3 to 4 times to facilitate the release of spermatozoa, after which the tissue was incubated at 37°C for a duration of 5 minutes to enable the sperm to disperse into the saline. Approximately 10 μl of the diluted sperm suspension was subsequently pipetted onto a blood counting chamber, and the sperm count was quantified under microscopic observation.

### 2.3 Detection of serum gonadal hormones

In accordance with the manufacturer’s prescribed protocols, we conducted enzyme-linked immunosorbent assays (ELISAs) to quantify the serum concentrations of key gonadal hormones. Specifically, the levels of testosterone, luteinizing hormone (LH), and follicle-stimulating hormone (FSH) were measured using the respective Mouse ELISA Kits (E-OSEL-M0003 for testosterone, E-EL-M3053 for LH, and E-EL-M0511 for FSH, all procured from Elabscience, China).

### 2.4 Measurement of GSH, oxidized glutathione (GSSG), catalase (CAT), and malondialdehyde (MDA)

The concentrations of GSH and GSSG in testicular tissues were measured using a dedicated quantification assay kit procured from Beyotime (Catalog No. S0053, Shanghai, China). Additionally, the activities of CAT and the levels of MDA were determined using the respective assay kits obtained from Jiancheng (Catalog No. A007-1-1, Nanjing, China) and Solarbio (Catalog No. BC0025, Beijing, China).

### 2.5 Quantitative determination of arachidonic acid (AA)

The tissue levels of AA were precisely quantified employing a murine-specific ELISA kit designed for the accurate measurement of AA (Catalog No. RJ17198, Renjie, Shanghai, China).

### 2.6 Iron concentration and distribution analysis

The level of ferrous iron (Fe^2+^) in the testicular tissue was quantitatively assessed using specialized assay kit (Catalog No. E-BC-K773-M, Beyotime, Shanghai, China). Furthermore, the histological distribution of iron ions within the testis was visualized using a Prussian Blue Iron Stain Kit (Enhanced with DAB, Catalog No. G1428, Solarbio, Beijing, China). Briefly, the testicular sample was fixed and embedded with paraffin to make a 5μm section. These sections were subjected to staining with Perl’s working solution at 37°C for 20 minutes, followed by rinsing with distilled water. Subsequently, the sections were treated with an incubation working solution, incubated at 37°C for an additional 20 minutes, and finally stained with an enhanced working solution for 15 minutes. The spatial distribution of iron ions was then examined and documented using a high-resolution digital scanning microscope (Nikon, Japan).

### 2.7 Western blot analysis

Testicular tissue samples were homogenized in pre-chilled radio-immunoprecipitation assay (RIPA) buffer, and the protein extracts were obtained by centrifugation at 12,000×g for 15 minutes at 4°C. The protein samples were then fractionated on 10% sodium dodecyl sulfate-polyacrylamide gel electrophoresis (SDS-PAGE) and transferred to polyvinylidene difluoride (PVDF) membranes. Non-specific binding was minimized by blocking the membranes with a 5% solution of non-fat dried milk in Tris-buffered saline (TBS) (pH 7.2) for 1 hour at room temperature. Subsequently, the membranes were incubated overnight at 4°C with the following antibodies: anti-KEAP1 (1:500, A1820, Abclonal, China), anti-NAD(P)H: quinone oxidoreductase (NQO-1) (1:500, A23486, Abclonal, China), anti-SLC7A11 (1:500, A2413, Abclonal, China), anti-SLC40A1 (FPN) (1:500, A14885, Abclonal, China), anti-Ferritin Heavy Chain (FTH1) (1:500, A19544, Abclonal, China), anti-Acyl-CoA Synthetase Long-Chain Family Member 4 (ACSL4) (1:500, A6826, Abclonal, China), anti-Zonula Occludens-1 (ZO-1) (1:500, A0659, Abclonal, China), anti-Occludin (1:1000, A2601, Abclonal, China), anti-p53 (1:1000, A0263, Abclonal, China), anti-SQSTM1/p62 (1:1000, A7758, Abclonal, China), anti-LC3B (1:1000, BM4827, Boster, China), anti-IFN-γ (1:1000, A00393-3, Boster, China), anti-cleaved-caspase-3 (9664S, Cell Signaling, Beverly, MA, USA), anti-HO-1 (1:1000, A1346, Abclonal, China), anti-GPX4 (1:1000, 59735S, Cell Signaling, Beverly, MA, USA), anti-NOD-like receptor protein 3 (NLRP3) (1:1000, 15101, Cell Signaling, Beverly, MA, USA), anti-Cyclooxygenase-2 (COX2) (1:1000, 12282T, Cell Signaling, Beverly, MA, USA), anti-CD71/TFR (1:1000, 13113, Cell Signaling, Beverly, MA, USA), anti-NRF2(1:1000, 12721T, Cell Signaling, Beverly, MA, USA). After removal of unbound antibodies using TBS containing 0.05% Tween 20, the membranes were incubated with the corresponding secondary antibodies for 1 hour at room temperature. β-actin served as an internal control to normalize the protein loading. The antigen-antibody complexes were visualized using an ultra-sensitive enhanced chemiluminescence (ECL) (NCM Biotech). The immunoreactive bands were quantified by densitometry using Image J software, a method previously validated in the literature (57).

### 2.8 Immunohistochemistry

Sections of paraffin-embedded testicular tissue, 5μm in thickness, were prepared sequentially. Following rehydration, antigen retrieval was executed via microwave. The immunohistochemical staining procedure was carried out utilizing a 3,3’-diaminobenzidine (DAB) substrate kit (Catalog No. G1212, Servicebio, Wuhan, China). The protocol included the inhibition of endogenous peroxidase activity in the tissue sections using a 3% hydrogen peroxide solution for 25 minutes, succeeded by rinsing in PBS three times. Subsequently, antigenic sites were demarcated using a 3% bovine serum albumin solution for 30 minutes, after which the sections were incubated with the primary antibodies at 37°C for an overnight period. The primary antibodies utilized were as follows: GPX4 (1:500, GB114327), and NRF2 (1:1000, GB113808), both procured from Servicebio, Wuhan, China. On the subsequent day, the sections underwent incubation with the corresponding secondary antibodies followed by the application of DAB. Subsequently, the sections were counterstained with hematoxylin for a minute. The immunostained slides were visualized and captured using a digital scanning microscope at a magnification of 200×.

### 2.9 Quantitative real-time polymerase chain reaction (qRT-PCR)

Total Ribonucleic Acid (RNA) was extracted with TRIzol reagent (T9424, Sigma-Aldrich, USA). Subsequently, the reverse transcription process was executed in accordance with the protocols provided by the manufacturer, utilizing the Takara cDNA Reverse Transcription Kits (Catalog number RR036a, TaKaRa, China). The resulting complementary DNA (cDNA) was then subjected to quantitative PCR (qPCR) on the quantitative PCR System (Model CFX384, BIO-RAD, USA). The relative quantification of mRNA expression was normalized to β-actin, and the data analysis was performed using GraphPad Prism 8.0 software. The sequences of the primers utilized in this study are delineated in **Supplementary Table 1**.

### 2.10 Analysis of T cells in testicular tissue via flow cytometry

Fresh mouse testes and spleens were meticulously dissected and immediately immersed in ice-cold RPMI 1640 medium (Catalog No. 10491, Solarbio, Beijing, China). The testes were further dissected and minced using scissors. The minced tissue was subjected to enzymatic digestion in a solution of RPMI 1640 supplemented with 0.5 mg/ml type IV Collagenase (Sigma), 0.1 mg/ml DNase I (Sigma-Aldrich), and 2% FBS at 37°C with agitation at 180 rpm for 20 minutes. Digestion was stopped by the addition of 1 ml of neutralization buffer, comprising RPMI 1640 with 2% FBS and 5 mM EDTA. The cell pellet was obtained via centrifugation (1,200g for 4min at 4°C) and subsequently resuspended in FACS buffer (PBS containing 2% FBS), filtered through a 100μm cell strainer and counted.

The cell suspensions were incubated with the following for 30 minutes on ice: Anti-mouse CD45-PE (BioLegend, Clone 30-F11), anti-mouse CD3-FITC (BioLegend, Clone 17A2), anti-mouse CD4-PerCP/Cy5.5 (BioLegend, Clone RM4-5), anti-mouse CD8α-Brilliant violet 421 (BioLegend, Clone 53-6.7). FACS buffer served as the diluent for all staining procedures. Then, they were transferred to round-bottom polypropylene FACS tubes. Data were collected using a BD LSR Fortessa X-20 cell analyzer (BD Biosciences), and subsequent analysis was conducted using FlowJo software version 10.

### 2.11 Detection of reactive oxygen species (ROS) in testicular tissue via flow cytometry

Testicular tissue, freshly excised, was meticulously collected and rinsed in ice-cold PBS to eliminate residual blood or debris. Subsequently, the tissue underwent mincing followed by enzymatic digestion utilizing a collagenase solution, facilitating cellular dissociation. The cell suspension was strained through a sterile mesh to achieve a homogeneous single-cell suspension. This suspension was then subjected to incubation with a ROS-sensitive fluorescent probe, 2’,7’-dichlorodihydrofluorescein diacetate (DCFH-DA), at a temperature of 37°C for 30 minutes. After staining, the cells underwent a series of washes with PBS to remove unreacted probe, followed by routine collection procedures. The cells were resuspended in PBS, mixed gently, and filtered through a 100μm cell strainer into designated tubes for immediate flow cytometric analysis. Data were collected using a BD LSR Fortessa X-20 cell analyzer (BD Biosciences), and subsequent analysis was conducted using FlowJo software version 10.

### 2.12 TUNEL assay

The TUNEL cell apoptosis assay kit (Catalog No. G1502, Servicebio, Wuhan, China) was used to detect cell apoptosis in testis tissue sections. In detail, the sections were subjected to deparaffinization and gradient ethanol treatment. The sections were fixed with 4% paraformaldehyde. After digestion with proteinase at 37°C for 10 minutes, the sections were then incubated with terminal deoxynucleotidyl transferase and digoxigenin-labeled dUTP (DIG-dUTP) at 37°C for 2 hours, marking the 3′-OH end of fragmented DNA with DIG-dUTP. The sections were blocked with 5% BSA blocking solution at room temperature for 30 minutes. Subsequently, the sections were incubated with biotinylated digoxigenin antibody (diluted with SABC) at 37°C for 30 minutes, followed by washing three times, each time for 5 minutes. Additionally, the sections were incubated with SABC-FITC at 37°C for 30 minutes, followed by 2-(4-Amidinophenyl)-6-indolecarbamidine dihydrochloride (C1005, Beyotime) staining at room temperature for 5 minutes. After staining, the tissue sections were sealed with an anti-quenching mounting medium. The stained tissues were observed under an Olympus BX53 microscope, and TUNEL-positive cells emitted red fluorescence.

### 2.13 Statistical analysis

The data were presented as the mean values accompanied by the standard error of the mean (SEM). To compare the statistical differences between two distinct groups, a Student’s t-test was employed in a two-tailed unpaired format. For the assessment of differences among more than two groups, a one-way analysis of variance (ANOVA) was conducted, followed by *post-hoc* testing using Fisher’s Least Significant Difference (LSD) method. Statistical significance was established at the threshold of P < 0.05. The aforementioned statistical analyses were conducted using GraphPad Prism software version 10.1.0.

## 3 Results

### 3.1 Effects of anti-PD-1 and RSV on organ weight, sperm concentration, serum gonadal hormone levels and the blood-testosterone barrier (BTB) integrity

The testicular coefficient, a critical indicator of testicular health, was ascertained by calculating the ratio of testicular weight to body weight. A statistical analysis was conducted among the treatment groups, evaluating parameters including body weight, testicular weight, and testicular coefficient. Upon analysis, it was observed that the administration of anti-PD-1, either as monotherapy or in combination with RSV, did not result in statistically significant differences in body weight, testicular weight, or testicular coefficient when compared to the control group. The results of these analyses were systematically detailed in **Supplementary Table 2**, provide valuable insights into the impact of these treatments on testicular health.

**Figure 1:**
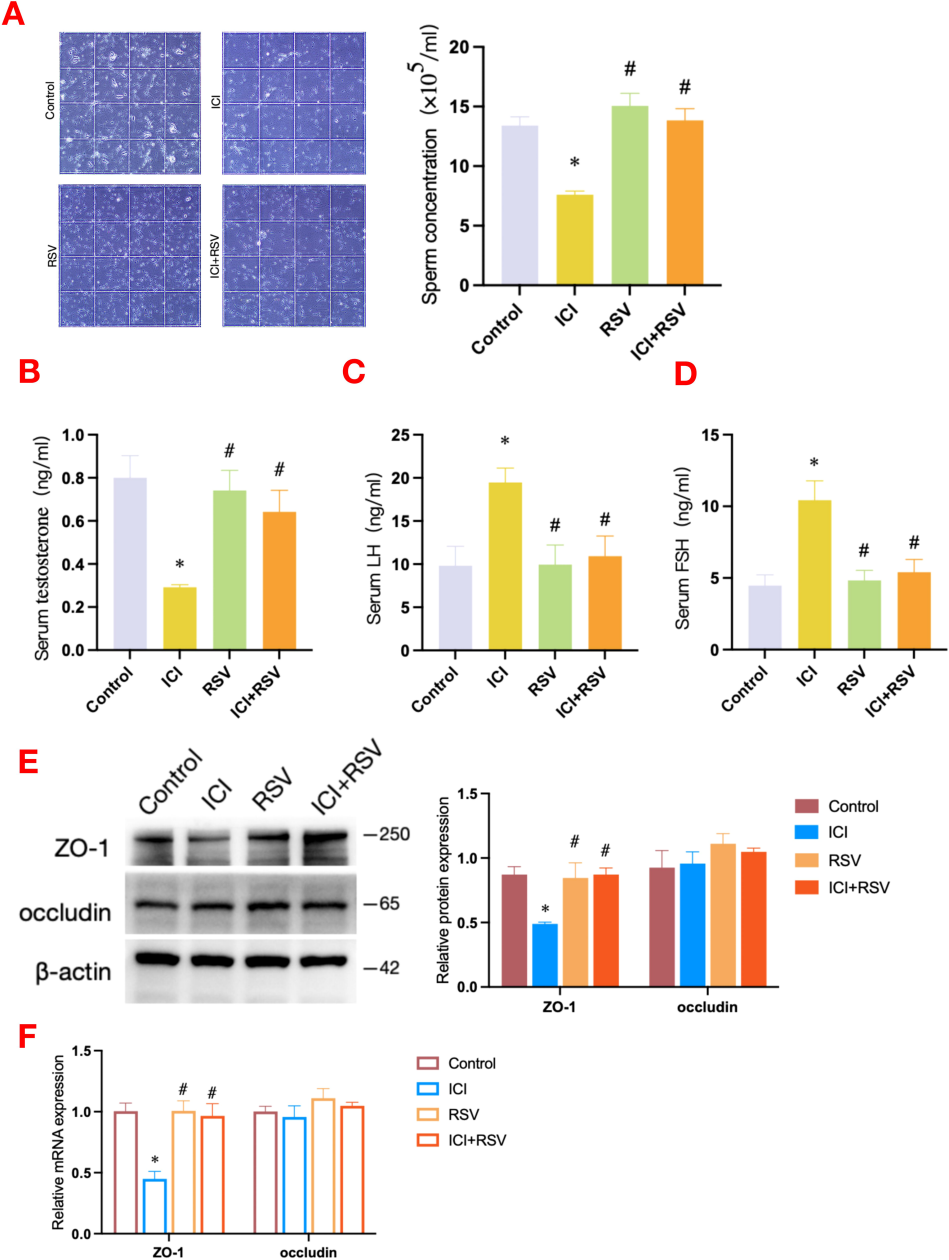
Effects of anti-PD-1 and RSV on sperm concentration, serum hormone levels and BTB integrity. Following the conclusion of the treatment period, a comprehensive assessment of sperm concentration was conducted **(A)**, the serum concentrations of testosterone **(B)**, LH **(C)**, and FSH **(D)** were measured (n = 6 per group). The integrity of the BTB was assessed by western blot to determine the protein expression levels of ZO-1 and occludin **(E)**, and by qRT-PCR to evaluate the gene expression levels of ZO-1 and occludin **(F)** (n = 3 per group). Data are presented as means ± SEM. *P < 0.05 vs. Control group; #P < 0.05 vs. ICI group.

As shown in **Figure 1A**, compared to the control group, treatment with anti-PD-1 resulted in a significant decrease in sperm concentration. In contrast, the co-administration of the ICI with RSV significantly improved sperm concentration compared to the ICI group alone. These findings indicated that while treatment with anti-PD-1 significantly disrupted spermatogenesis, treatment with RSV ameliorated the adverse effects of anti-PD-1 on spermatogenesis in mice.

The appropriate secretion of hormones is integral to the maintenance of normal spermatogenesis. In order to elucidate the toxic effects of anti-PD-1 on murine testicular function, we quantified serum hormone levels, including testosterone, LH, and FSH. As shown in **Figure 1B**, testosterone level was significantly reduced in the ICI group compared to the control group. Meanwhile, as illustrated in **Figures 1C, D**, LH and FSH levels were significantly increased in the ICI group when compared to the control group. Notably, the testosterone level in the ICI+RSV treatment group was significantly elevated (**Figure 1B**), positioning it between the levels observed in the control and ICI groups. In contrast, the LH and FSH levels in the ICI+RSV group were significantly decreased (**Figures 1C, D**), placing them between the levels found in the control and ICI groups. Collectively, these results indicated that treatment with anti-PD-1 disrupted the gonadal hormones levels. The application of RSV was found to ameliorate the deleterious effects of anti-PD-1 on hormones.

The BTB, which is composed of Sertoli and germ cells, creates a specialized microenvironment crucial for the proper development of spermatids. In order to evaluate the impact of anti-PD-1 on BTB integrity, we examined the expression profiles of proteins and genes associated with the BTB across various experimental groups. A significant reduction in the expression level of the tight junction (TJ) protein ZO-1 was observed in the ICI group when compared to the control group, as depicted in **Figure 1E**. In contrast to the ICI group, the ICI+RSV treatment group exhibited a significant upregulation of both the protein and mRNA expression levels of ZO-1, as depicted in **Figures 1E, F**. Additionally, there were no significant differences in the protein and mRNA expression levels of occludin among various groups, as shown in **Figures 1E, F**. These observations suggested that anti-PD-1 may potentially induce testicular impairment by affecting the BTB integrity. Moreover, treatment with RSV seems to mitigate the BTB disruption induced by anti-PD-1.

### 3.2 Effects of anti-PD-1 and RSV on T cells in testicular tissue

**Figure 2:**
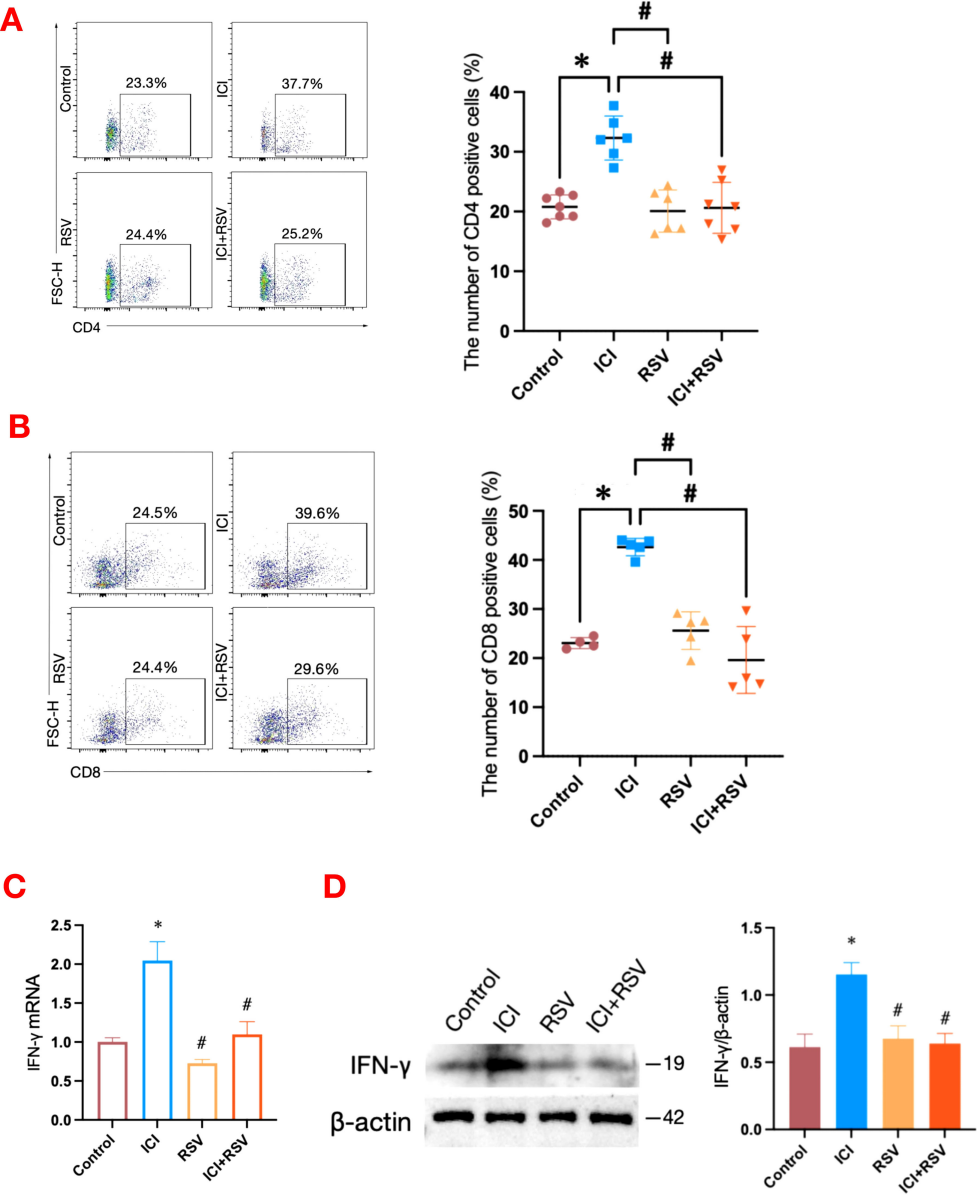
Effects of anti-PD-1 and RSV on T cells and IFN-γ in testicular tissue. Percentages of testicular T cells, including CD4^+^ T cells **(A)** and CD8^+^ T cells **(B)**, were analyzed by flow cytometry in tumor-bearing mice after the final treatment (n = 4 at least, in each group). The mRNA level of IFN-γ in the testis was analyzed by qRT–qPCR **(C)** and the protein level of IFN-γ was analyzed by western blot **(D)** (n = 3 per group). Data are presented as means ± SEM. *P < 0.05 vs. Control group; #P < 0.05 vs. ICI group.

Given that ICIs are designed to stimulate and enhance immune responses, we conducted a flow cytometry analysis to assess the T immune cells within the testes of tumor-bearing mice. The proportion of CD4^+^ T cells (Control: 20.79 ± 2.01%, ICI: 32.30 ± 3.67%, P<0.0001, **Figure 2A**) and CD8^+^ T cells (Control: 23.05 ± 1.12%, ICI: 42.62 ± 1.78%, P<0.0001, **Figure 2B**) within the testes were significantly increased in the ICI group compared to the control group. Furthermore, the co-administration of RSV with ICI significantly reversed these increases, reducing the proportion of CD4^+^ T cells (ICI: 32.30 ± 3.67%, ICI+RSV: 20.61 ± 4.27%, P<0.0001, **Figure 2A**) and CD8^+^ T cells (ICI: 42.62 ± 1.78%, ICI+RSV: 19.62 ± 6.81%, P<0.0001, **Figure 2B**) compared to the ICI group alone. These observations indicated that anti-PD-1 enhanced T cell-mediated immune responses within the testes of tumor-bearing mice, and highlighted the potential of RSV to modulate the T cell-mediated immune responses induced by anti-PD-1 treatment, suggesting a possible therapeutic strategy to mitigate the immunological effects of ICIs in testicular tissue.

In addition to expanding immune cell populations, ICIs are known to upregulate systemic cytokine levels in patients receiving these treatments. The blockade of PD-1 significantly increased testicular interferon-gamma (IFN-γ) mRNA level following treatment with anti-PD-1 (**Figure 2C**). To evaluate intra-testicular IFN-γ protein level, western blot analysis was performed on whole testicular tissue collected from tumor-bearing mice after the final treatment. The protein expression of IFN-γ was upregulated in the testes of the ICI group compared to the control group (**Figure 2D**). In contrast, the protein and mRNA levels of IFN-γ in the ICI+RSV group were significantly decreased (**Figures 2C, D**). Collectively, these data indicated that a local inflammatory response in the testis occurred following anti-PD-1 treatment, potentially contributing to the disruption of the BTB. Moreover, treatment with RSV appears to ameliorate the disruption caused by anti-PD-1.

### 3.3 Effects of anti-PD-1 and RSV on testicular oxidative stress state and antioxidant defense system

**Figure 3:**
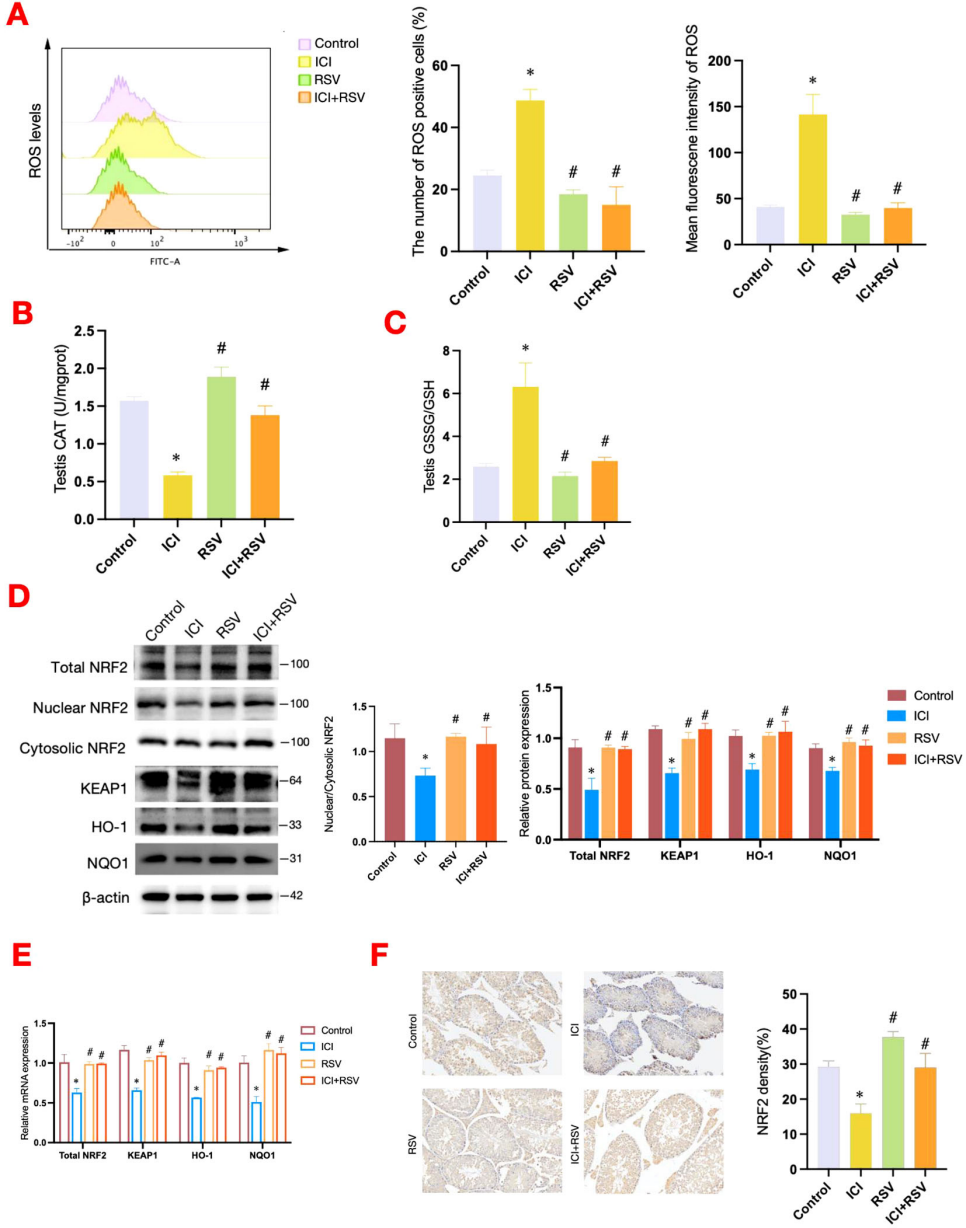
Effects of anti-PD-1 and RSV on testicular oxidative stress and antioxidant defense system. At the conclusion of the treatment period, the level of ROS within testicular tissue was analyzed by flow cytometry in tumor-bearing mice (n = 3 per group) **(A)**. The activities of CAT in testicular tissues (n=6 per group) **(B)**, the ratio of GSH/GSSG in testicular tissues (n=6 per group) **(C)** were determined. The NRF2-KEAP1 signaling pathway was assessed at the protein level by western blot **(D)** and at the gene level by qRT-PCR **(E)** (n = 3 per group). **(F)** Representative images of immunohistochemistry staining for NRF2 in testicular tissue from each group, accompanied by quantification at 200× magnification. Data are presented as means ± SEM. *P < 0.05 vs. Control group; ^#^P < 0.05 vs. ICI group.

Oxidative damage is a prevalent cause of reproductive system impairment. To explore the impact of anti-PD-1 on testicular oxidative stress status, we utilized flow cytometry to measure ROS levels within testicular tissue. Additionally, we assessed the activity of CAT and the ratio of GSH/GSSG using standardized assay kits. The ROS levels were significantly increased in the ICI group compared to the control group, as depicted in **Figure 3A**. Concurrently, the ICI group exhibited a significant decrease in CAT activity and the GSH/GSSG ratio, as shown in **Figures 3B, C**. Treatment with RSV mitigated these detrimental effects, significantly reducing ROS levels and enhancing CAT activity and the GSH/GSSG ratio compared to the ICI group (**Figures 3A–C**). Collectively, these results suggested that anti-PD-1 could disrupt testicular antioxidant defenses, leading to an oxidative stress in testis. The beneficial effects of RSV highlighted its potential in restoring redox balance.

The NRF2-KEAP1 signaling pathway is essential for maintaining redox homeostasis and plays a pivotal role in the regulation of oxidative stress through the activation of downstream transcription. The ratio of nuclear NRF2 to cytoplasmic NRF2 is an important index for assessing the transcriptional activity of NRF2. In this study, ICI treatment significantly inhibited the NRF2 signaling pathway, as evidenced by decreased total protein expression levels of NRF2, reduced nuclear-to-cytoplasmic NRF2 ratio, and diminished protein and gene expression levels of KEAP1, HO-1, and NQO1 (**Figures 3D, E**). Conversely, treatment with RSV significantly upregulated these parameters, enhancing NRF2 signaling and antioxidant gene expression (**Figures 3D, E**). Immunohistochemical analysis confirmed these findings, demonstrating decreased NRF2 expression in testicular tissue of the ICI group, with a notable increase expression in the ICI+RSV group (**Figure 3F**). Furthermore, the decreased nuclear expression of NRF2 was observed in the ICI group relative to the control group (**Figure 3F**).

In summary, these findings indicated that treatment with anti-PD-1 suppressed the NRF2 signaling pathway and induced oxidative stress in testicular tissue. Conversely, treatment with RSV activated the NRF2 pathway and mitigated the oxidative stress in the reproductive system caused by anti-PD-1.

### 3.4 Effects of anti-PD-1 and RSV on testicular iron homeostasis

**Figure 4:**
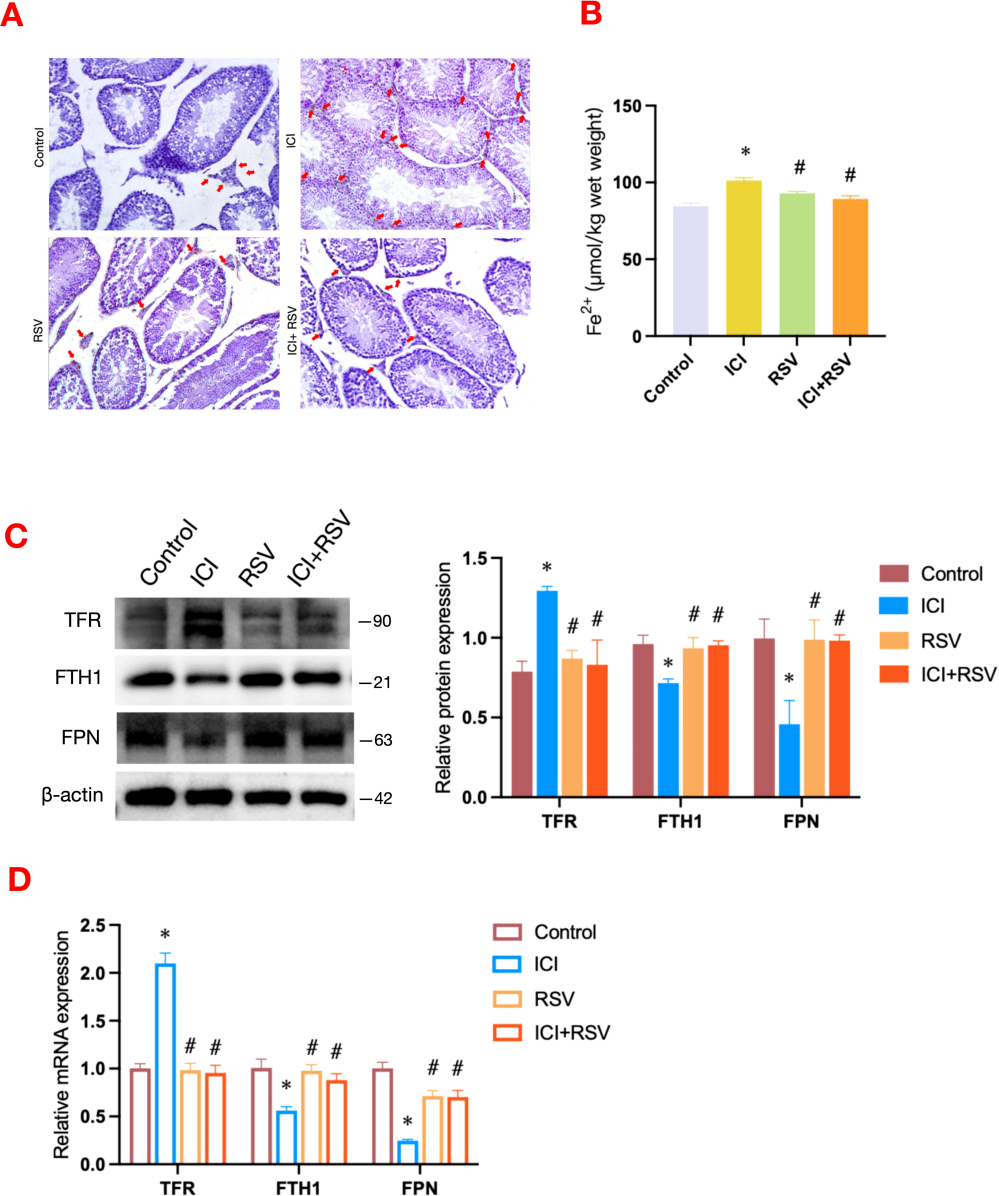
Effects of anti-PD-1 and RSV on testicular iron homeostasis. The distribution and quantification of Fe^2+^ in testicular tissue across different treatment groups were visualized using DAB-enhanced Perl’s Prussian blue staining **(A)**. Scale bar = 20 μm. Following the treatment period, the level of Fe^2+^ within the testes was measured (n=6 per group) **(B)**. Iron metabolism-related pathway was examined by western blot assay for the protein expressions **(C)** and by qRT-PCR assay for the gene expressions **(D)** (n = 3 per group). Data are presented as means ± SEM. *P < 0.05 vs. Control group; ^#^P < 0.05 vs. ICI group.

Iron homeostasis is essential for sustaining testosterone synthesis and spermatogenesis (13). To elucidate the impact of anti-PD-1 on testicular iron homeostasis, we analyzed testicular iron distribution and content, as well as the expression of iron transport-related factors. The control group exhibited a scattered iron distribution (**Figure 4A**). In contrast, the ICI group showed notable iron deposition in the testicular interstitium (**Figure 4A**). This deposition was significantly reduced in the ICI+RSV group compared to the ICI group (**Figure 4A**). Consistent with the DAB-enhanced Perl’s Prussian blue staining results, the level of Fe^2+^ was significantly elevated in the ICI group relative to the control group (**Figure 4B**), indicating iron overload induced by anti-PD-1 in the testis. In comparison to the ICI group, the level of Fe^2+^ was significantly reduced in the ICI+RSV groups, as shown in **Figure 4B**.

Iron overload is associated with dysregulation of iron metabolism, encompassing iron uptake, storage, and release. Subsequently, we employed western blot and qRT-PCR to assess the expression levels of factors involved in iron transport and storage. Compared to the control group, the expression levels of TFR, an indicator of iron uptake, were upregulated at both the gene and protein levels in the ICI group, as shown in **Figures 4C, D**. These upregulations were effectively reversed in the ICI+RSV group (**Figures 4C, D**). Relative to the control group, the expression levels of FTH1, associated with iron storage, and FPN, involved in iron release, were decreased in the ICI group (**Figures 4C, D**). Conversely, the protein and gene expression levels of FTH1 and FPN were significantly upregulated in the ICI+RSV group compared to the ICI group (**Figures 4C, D**).

The collective data indicated that administration of anti-PD-1 enhanced iron transfer into cells and diminished the ability of cells to stabilize and export iron ions, leading to testicular iron overload. This suggested that anti-PD-1 disrupted testicular iron homeostasis. Furthermore, the application of RSV ameliorated the disruption of iron homeostasis induced by anti-PD-1.

### 3.5 Effects of anti-PD-1 and RSV on testicular lipid peroxidation

**Figure 5:**
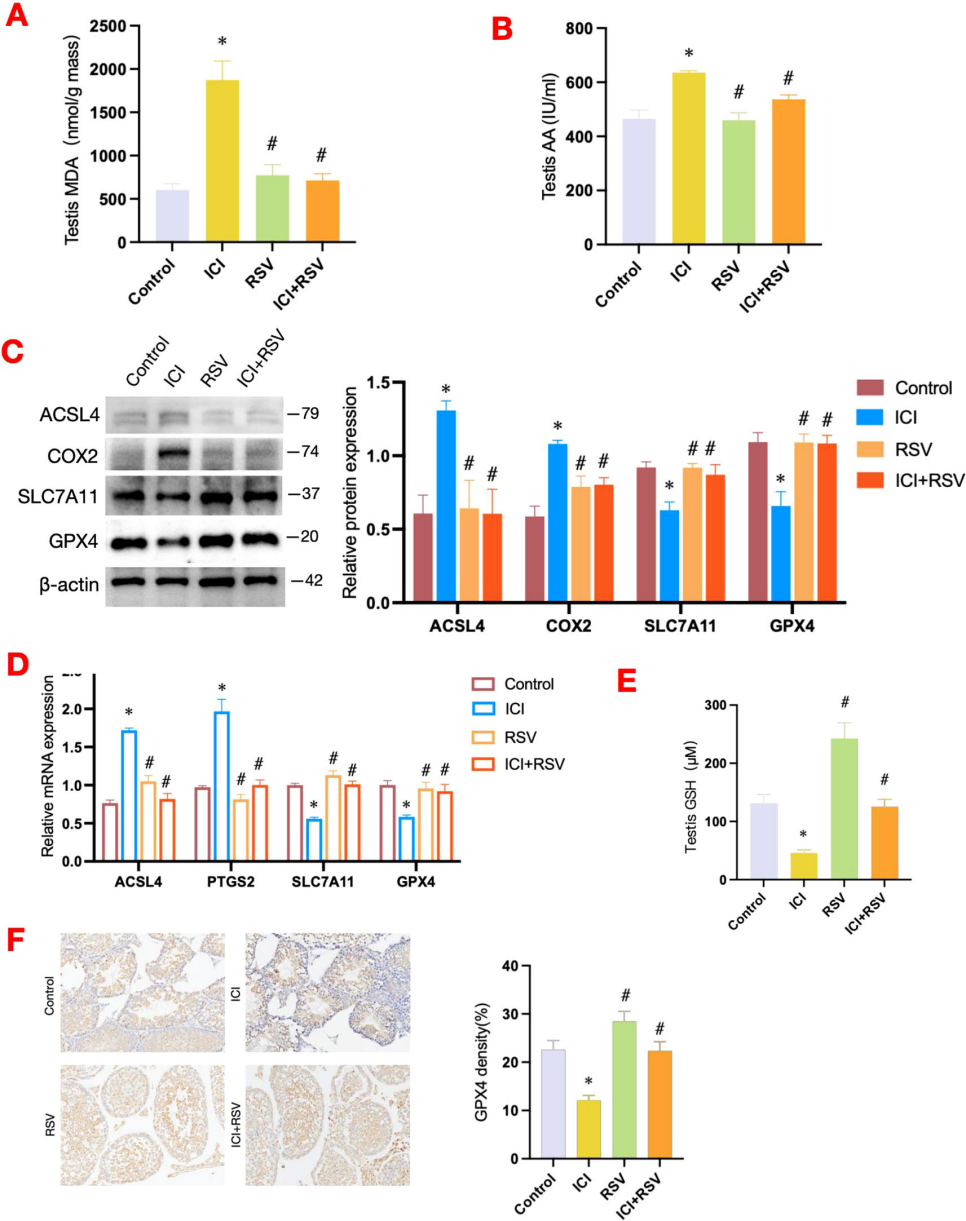
Effects of anti-PD-1 and RSV on ferroptosis-related pathways. Following the conclusion of the treatment period, the MDA level within testes was quantified (n=6 per group) **(A)**, alongside the measurement of AA content (n=6 per group) **(B)**. Ferroptosis-related pathway was examined by western blot assay for the protein expressions **(C)** and by qRT-PCR assay for the gene expressions **(D)** (n = 3 per group). At the conclusion of the treatment period, the concentration of GSH in testicular tissue was measured using a dedicated quantification assay kit (n=6 per group) **(E)**. **(F)** Representative images of immunohistochemistry staining for GPX4 in testicular tissue from each group, accompanied by quantification at 200× magnification. Data are presented as means ± SEM. *P < 0.05 vs. Control group; ^#^P < 0.05 vs. ICI group.

Lipid peroxidation is a key biological process involved in ferroptosis, and MDA, a byproduct of this process, serves as a biomarker for oxidative stress and ferroptosis in tissues (54). In this study, we observed a significant increase in testicular MDA level in the ICI group compared to the control group (**Figure 5A**). Additionally, we focused on the AA metabolic pathway, recognizing its pivotal role in representing the biochemical cascade of lipid peroxidation production. A significant elevation in AA content was observed in the ICI group compared to the control group (**Figure 5B**). Notably, the levels of MDA and AA were substantially reduced in the ICI+RSV group compared to the ICI group (**Figures 5A, B**).

Compared to the control group, the protein and mRNA expression levels of key enzymes involved in the lipid peroxidation metabolic pathway, including ACSL4 and COX2, were also upregulated following treatment with anti-PD-1 (**Figures 5C, D**). Conversely, in the ICI+RSV group, the protein and mRNA levels of ACSL4 and COX2 were markedly decreased compared to the ICI group (**Figures 5C, D**).

Altogether, these findings suggested that anti-PD-1 may activate specific factors associated with lipid oxidation metabolic pathways, leading to an accumulation of lipid peroxides within the testes of mice. Fortunately, treatment with RSV mitigated the accumulation of lipid peroxides induced by anti-PD-1.

### 3.6 Effects of anti-PD-1 and RSV on the testicular SLC7A11-GSH-GPX4 axis

The SLC7A11-GSH-GPX4 signaling axis is recognized as a canonical defense mechanism against ferroptosis. SLC7A11, a key component of the system Xc-, facilitates the uptake of extracellular cystine in exchange for glutamate (18). Once inside the cell, cystine is converted to cysteine, which is essential for the synthesis of GSH. GSH acts as a co-factor for GPX4, enabling it to reduce lipid peroxidation and thereby prevent ferroptosis (19–21). Our results revealed a significant decrease in GSH level in the ICI group in contrast to the control group (**Figure 5E**). Consequently, we further investigated the impact of anti-PD-1 on the testicular SLC7A11-GSH-GPX4 axis. Compared to the control group, both the protein and mRNA expression levels of SLC7A11 and GPX4 were significantly downregulated in the ICI group (**Figures 5C, D**). Notably, the protein and gene expression levels of SLC7A11 and GPX4 were substantially increased in the ICI+RSV group compared to the ICI group (**Figures 5C, D**). Consistent with these results, immunohistochemical analysis revealed a significant reduction in GPX4 expression in the testicular tissue following anti-PD-1 treatment, which was reversed by the supplementation of RSV (**Figure 5F**).

Taken together, our results suggested that anti-PD-1 may induce damage to the SLC7A11-GSH-GPX4 pathway, which could be ameliorated by the administration of RSV.

## 4 Discussion and perspective

**Figure 6:**
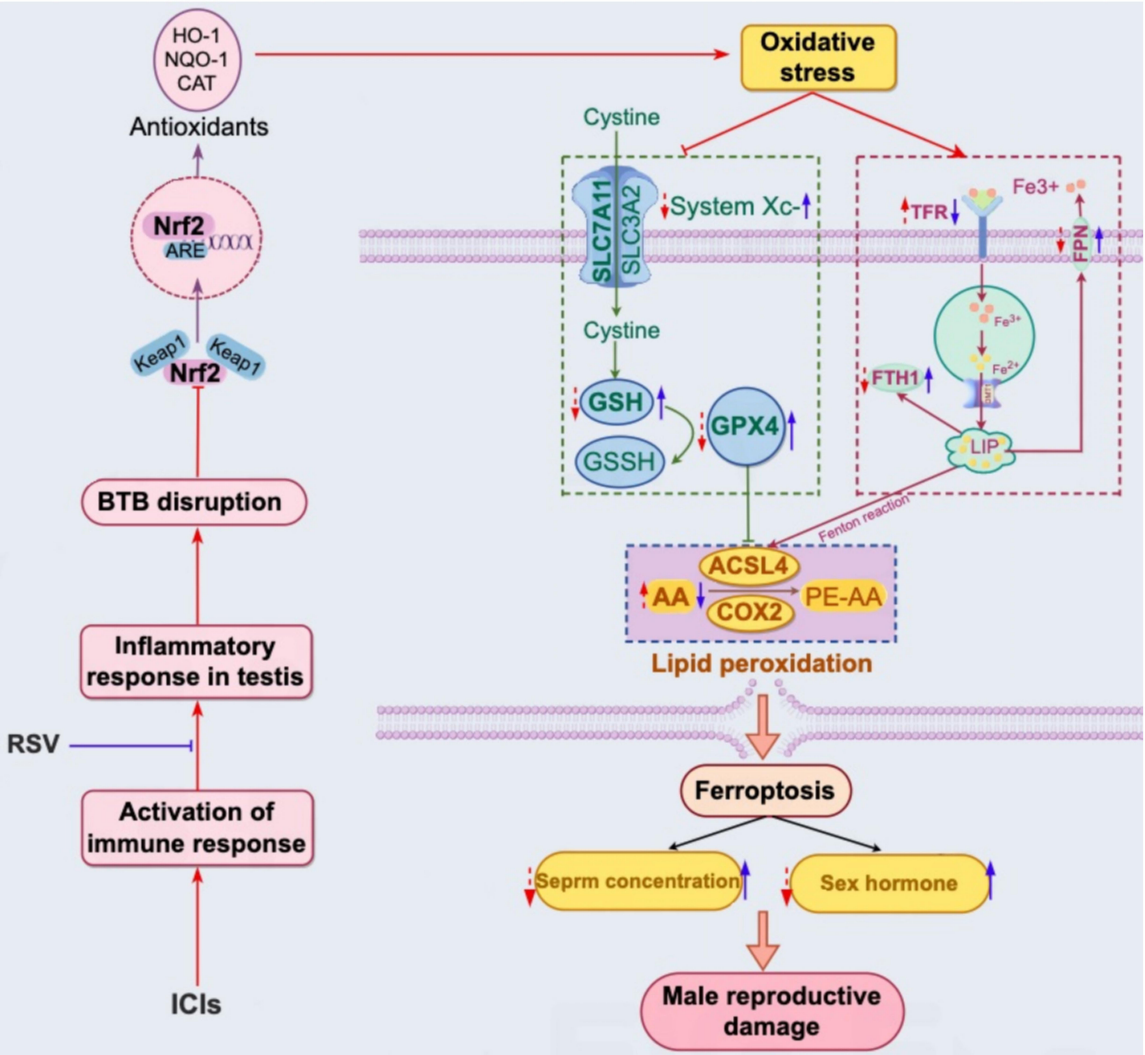
Schematic representation of the proposed mechanism for reproductive damage induced by ICIs through testicular inflammation, oxidative stress, and ferroptosis, as well as the protective role of RSV against ICIs-induced reproductive toxicity. ICIs enhance T cell-mediated immune responses and increase systemic cytokine levels, particularly IFN-γ, within the testis. This leads to a localized inflammatory response, potentially contributing to the disruption of the BTB and an imbalance between oxidative and antioxidant systems, such as the NRF2-KEAP1 pathway and common antioxidant enzymes. Consequently, oxidative stress in the testis may result from these imbalances, acting as an inducer for iron homeostasis disorder, lipid peroxidation, and the subsequent induction of ferroptosis. The purple wireframe on the right illustrates the application of ICIs (indicated by red arrows), which induces downregulation of FTH1 and PFN expression, and upregulation of PFN expression, leading to iron overload in testicular tissue. Furthermore, ICIs exacerbate lipid peroxidation through the upregulation of lipid metabolic enzymes (ACSL4 and COX2) (red arrows in the blue wireframe below) and the inhibition of SLC7A11, GSH, and GPX4 expression (red arrows in the green wireframe on the left). Significantly, RSV can counteract ICIs-induced reproductive toxicity by reducing testicular inflammation, activating the NRF2 signaling pathway, and maintaining iron and lipid homeostasis (blue arrows).

In our comprehensive *in vivo* study using tumor-bearing mice, we observed significant testicular damage following treatment with anti-PD-1. Specifically, we found that anti-PD-1 treatment resulted in a marked reduction in sperm concentration, disturbances in gonadal hormones levels and disruptions in the integrity of BTB. Additionally, our data revealed a substantial increase in T cells infiltration and inflammatory cytokine expression in the testes of mice treated with anti-PD-1. Importantly, to our knowledge, this study is the first to suggest that anti-PD-1 induces oxidative stress, which subsequently triggers ferroptosis in the testis. Furthermore, we discovered that RSV protects against anti-PD-1-induced testicular oxidative stress and ferroptosis. The potential mechanisms underlying the testicular impairment induced by anti-PD-1 and the protective role of RSV are illustrated and discussed in detail in **Figure 6**.

Previous clinical research has indicated that a subset of patients may experience impaired spermatogenesis (58–60) and even azoospermia (60) following treatment with ICIs. In addition, abnormally low testosterone levels have been documented in some cases after ICIs treatment (61, 62). Similarly, our experimental observations in mice showed a reduction in sperm concentration and testosterone level. Furthermore, we noted an increase in LH and FSH levels, which may suggest the potential onset of primary gonadal dysfunction following anti-PD-1 administration. Although further studies are required to confirm this association, our preliminary findings highlight the need for consideration in long-term patient management. To date, only one case of primary gonadal dysfunction has been reported (63). The low prevalence of this condition may be attributed to insufficient evaluation methods to detect such patients, along with a high incidence of underreporting due to patient privacy concerns and the relatively mild severity of the disease. This study indicated that anti-PD-1 can induce testicular damage, as evidenced by decreased sperm concentration and disruption of gonadal hormones levels.

The testis plays a critical role in establishing and maintaining a protected environment essential for the growth and maturation of germ cells (64). The BTB is crucial for this microenvironment and is maintained by proteins such as ZO-1, which is found in TJ and facilitates the passage of materials (65). The disruption of BTB integrity, a key finding of our study, suggested that anti-PD-1 treatment may have altered the testicular microenvironment, thereby affecting spermatid development and maturation. The downregulation of TJ proteins like ZO-1, along with the upregulation of inflammatory cytokine and T cells infiltration, suggested an immune-mediated mechanism for BTB disruption induced by anti-PD-1. It is known that the expansion of effector T cells may overpower the suppressive mechanisms of regulatory T cells, provoking an autoimmune response and leading to impaired spermatogenesis, autoimmune orchitis, and/or azoospermia (66).

The oxidative stress, which refers to an imbalance between levels of ROS and antioxidants, is a primary cause of male infertility. While a small amount of ROS is necessary for sperm physiological functions, high levels of ROS can cause infertility not only through lipid peroxidation and DNA damage but also through the inactivation of enzymes and oxidation of proteins in spermatozoa (67, 68). The testis, with its high content of polyunsaturated membrane lipids, is particularly susceptible to oxidative stress. Oxidative stress can result from immature spermatozoa, inflammatory factors, genetic mutations, lifestyle factors, and altered gonadal hormone levels (67). In our study, oxidative stress emerged as a critical factor in testicular damage induced by anti-PD-1, characterized by elevated levels of ROS and diminished antioxidant capacity, as indicated by a reduction in CAT activity and the GSH/GSSG ratio. NRF2 is an antioxidant transcription factor that plays an essential role in maintaining redox balance (30, 31). Activated by a range of oxidative and electrophilic stimuli, including ROS, heavy metals, and certain disease processes, NRF2 mediates the induction of a spectrum of cyto-protective proteins. These proteins include phase II enzymes like NQO-1 and CAT, as well as antioxidant proteins such as HO-1, through the antioxidant response element (ARE) pathway (32). In our study, we further investigated whether anti-PD-1 induces oxidative stress in testicular tissue by inhibiting the NRF2 signaling pathway. We found that anti-PD-1 inhibited the total expression of NRF2, reduced the nuclear-to-cytoplasmic NRF2 ratio, and diminished the expression of KEAP1, HO-1, and NQO1. These results indicated that anti-PD-1 can reduce the expression of downstream antioxidant enzymes and disturb the antioxidant system by inhibiting the NRF2 signaling pathway.

Ferroptosis, a newly defined form of oxidative stress-mediated cell death, is strongly associated with ROS-mediated testicular damage (69, 70). However, its potential involvement in ICIs-induced reproductive toxicity has not been previously explored. Ferroptosis is primarily characterized by iron overload, depletion of GSH and GPX4, and lipid peroxidation (71). Our study demonstrated that anti-PD-1 led to iron accumulation in the testis, as confirmed by Perl’s Prussian blue staining and an increased level of Fe^2+^, indicating testicular iron overload. Iron homeostasis is modulated by a set of proteins, including transferrin for iron binding, TFR for iron uptake, ferritin for iron storage, and FPN for iron export. The TFR on the cell membrane recognizes the binding of iron to transferrin. Once inside the cell, iron is either utilized for metabolism or stored by ferritin. FPN, the only known mammalian cellular iron-export protein, reflects the cellular capacity to export iron. In our study, anti-PD-1 increased testicular TFR expression and decreased the expressions of FTH1 and FPN, indicating enhanced iron uptake and reduced iron stabilization and exportation, leading to testicular iron overload. Depletion of the SLC7A11-GSH-GPX4 system is a well-documented biochemical process associated with ferroptosis. Upregulation of SLC7A11 boosts GSH production, preventing the accumulation of lipid peroxidation products and inhibiting ferroptosis. Iron overload can inhibit SLC7A11 expression and trigger ferroptosis (72). GPX4, a GSH-dependent free radical scavenger (73), is essential for preventing ferroptosis, with its loss exacerbating ferroptosis-induced lethality (74). Consistent with these findings, our study found that anti-PD-1 increased testicular iron content, along with the inhibition of SLC7A11, and depletion of GSH and GPX4, indicating disruption of GSH biosynthesis through the SLC7A11-GPX4 antioxidant system and the triggering of ferroptosis.

Elevated levels of MDA, an indicator of lipid peroxidation, suggest that anti-PD-1 could induce lipid peroxidation in the testis. Lipid peroxidation, initiated by the reaction between polyunsaturated fatty acids and ROS in the phospholipid cell membrane, is another critical factor for ferroptosis activation. Intracellular iron from the Fenton reaction catalyzes the generation of hydroxyl radicals, promoting the peroxidation of membrane polyunsaturated fatty acids, such as AA and phosphatidylethanolamine, ultimately leading to cell ferroptosis (75). ACSLL4, a key initiator of ferroptosis, converts AA to arachidonoyl-CoA (AA-CoA), increasing ROS and precipitating ferroptosis (76, 77). Moreover, the biosynthesis of prostaglandin derivatives from AA is stringently regulated by the rate-limiting enzyme prostaglandin-endoperoxide synthase 2 (PTGS2, also known as COX2). The expression of COX2 is closely associated with intracellular lipid accumulation and has been identified as a downstream molecular marker of ferroptosis in various studies (20). Notably, the activation of the ACSL4-COX2 pathway promotes AA metabolism during ferroptosis (75, 78, 79), and depleting lipid peroxidation substrates by inhibiting ACSL4 activity suppresses ferroptosis sensitivity (80, 81). Consistent with these findings, our research discovered that anti-PD-1 increased the content of AA and the expression of key enzymes in the AA metabolic pathway, including ACSL4 and COX2, suggesting a potential mechanism by which anti-PD-1 might induce ferroptosis in the testis through the facilitation of lipid peroxidation via the AA metabolic pathway.

In addition to confirming that anti-PD-1 induced ferroptosis in testicular tissue, as depicted in **Supplementary Figure S1**, we investigated other forms of cell death, including apoptosis, autophagy and pyroptosis. Protein levels of cleaved-caspase-3 and p53, along with the TUNEL assay, provided no significant evidence of apoptosis in testicular tissue following anti-PD-1 treatment compared to the control group. Similarly, LC3B and p62, markers of autophagy, indicated no induction of autophagy in testicular tissue following anti-PD-1 treatment. Furthermore, NLRP3 expression did not significantly differ between the ICI group and the control group, suggesting that anti-PD-1 did not trigger pyroptosis. Consequently, our study suggests that anti-PD-1 primarily induces testicular damage through the ferroptosis pathway.

Furthermore, our study evaluated the efficacy of RSV in alleviating reproductive toxicity induced by ICIs. As a polyphenolic compound with numerous properties, RSV has been shown to modulate various cellular processes and protect against organ toxicity (44–55). In the context of anti-PD-1-induced testicular damage, RSV demonstrated significant therapeutic potential. As an anti-inflammatory agent, RSV improved the pro-inflammatory microenvironment in testicular tissue induced by anti-PD-1, characterized by reduced T cells infiltration and inflammatory cytokine level. It also reversed the disruption of BTB caused by anti-PD-1. Acting as an NRF2 agonist, RSV rectified the oxidative-antioxidative imbalance by upregulating the expression of NRF2, KEAP1, HO-1, and NQO1, along with enhancing common antioxidant enzymes such as CAT and GSH. These actions were further supported by our previous research and extensive studies demonstrating RSV’s positive effects in countering reproductive toxicity (32, 52–55). In addition to its anti-inflammatory and antioxidant properties, RSV also exhibited anti-ferroptosis potential by activating the SLC7A11-GSH-GPX4 system, regulating iron metabolism, and scavenging lipid peroxidation. The beneficial effects of RSV on hormonal levels and sperm concentration further confirm its potential as a therapeutic intervention for ICIs-induced damage to the male reproductive system.

In future studies, we plan to employ immunodeficient mice to confirm whether T cells infiltration directly causes the male reproductive toxicity induced by ICIs. Additionally, a central focus will be on determining if analogous effects occur in humans. We will further validate the pivotal role of NRF2 and GPX4 upregulation and activation in protecting the testis from anti-PD-1 using gene knockout mice. Extensive research is needed to uncover any unidentified mechanisms behind ICIs-induced male reproductive dysfunction. It is also crucial to elucidate the precise molecular mechanisms of RSV’s protective actions and to assess its clinical efficacy in alleviating reproductive toxicity caused by ICIs. Furthermore, exploring other natural compounds with comparable protective properties may lead to the development of therapeutic interventions to preserve the fertility of cancer patients receiving ICIs treatment.

## Data Availability

The original contributions presented in the study are included in the article/**Supplementary Material**. Further inquiries can be directed to the corresponding author.

## Supplementary material

Supplementary Figure 1 Effects of anti-PD-1 and RSV on other types of cell death. At the conclusion of the treatment period, common proteins associated with apoptosis (e.g., cleaved-caspase-3, p53), autophagy (e.g., LC3B, p62) and pyroptosis(e.g., NLRP3) were examined by western blot assay (n=3 per group) **(A)**. Testicular apoptosis also was examined by TUNEL staining for TUNEL-positive cells **(B)**.
