# Supplementary figures and images for: Resveratrol alleviates testicular toxicity induced by anti-PD-1 through regulating the NRF2-SLC7A11-GPX4 pathway
Source: Front Immunol. 2025 Mar 12;16:1529991. doi: 10.3389/fimmu.2025.1529991 (PMC11937136; doi:10.3389/fimmu.2025.1529991)

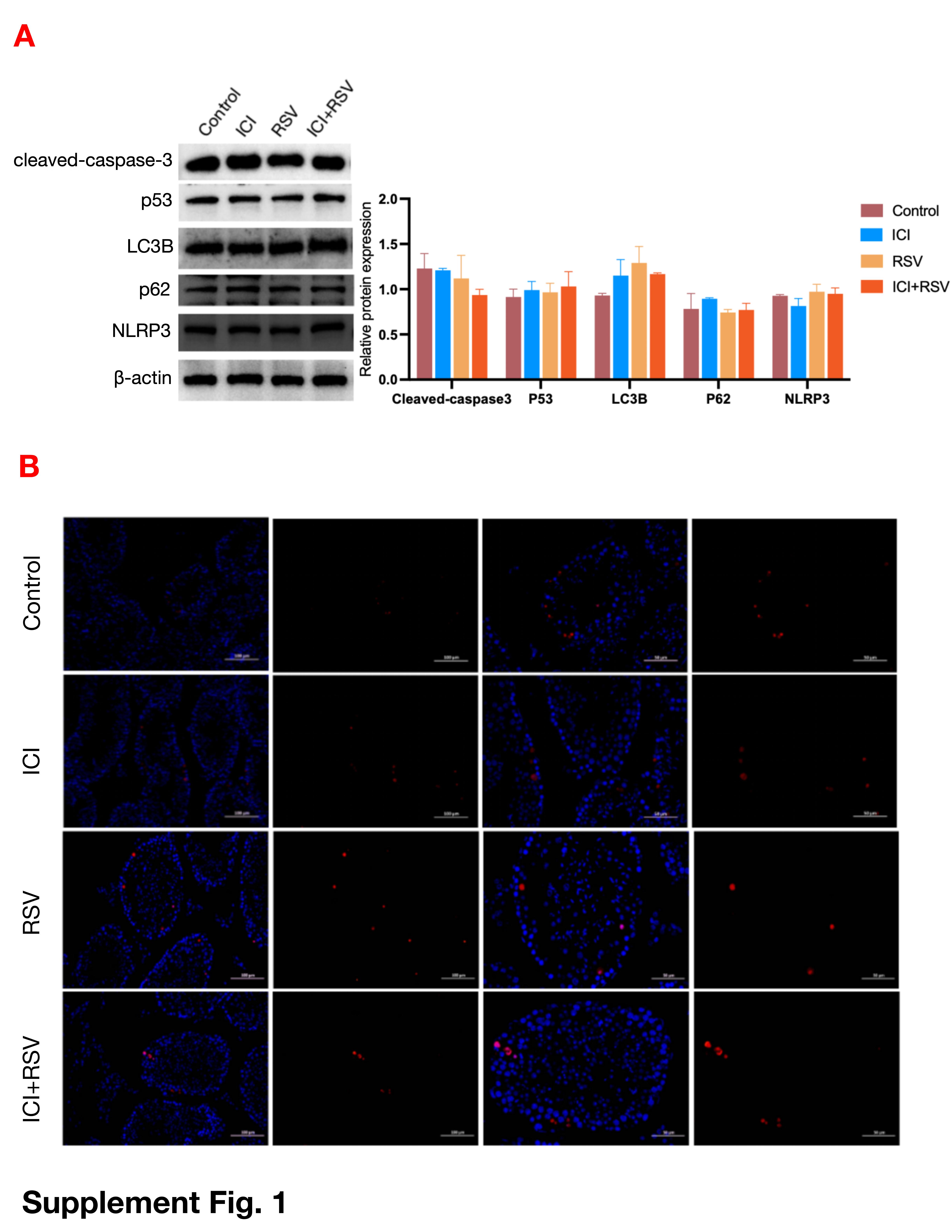

Supplement: Supplementary Figure 1 — Effects of anti-PD-1 and RSV on other types of cell death. At the conclusion of the treatment period, common proteins associated with apoptosis (e.g., cleaved-caspase-3, p53), autophagy (e.g., LC3B, p62) and pyroptosis(e.g., NLRP3) were examined by western blot assay (n=3 per group) (A). Testicular apoptosis also was examined by TUNEL staining for TUNEL-positive cells (B). [file Image1.jpeg]
